# Role of smartphone addiction in gambling passion and schoolwork engagement: a Dualistic Model of Passion approach

**DOI:** 10.1186/s40405-016-0018-8

**Published:** 2016-08-26

**Authors:** Ibeawuchi K. Enwereuzor, Leonard I. Ugwu, Dorothy I. Ugwu

**Affiliations:** 1 Department of Psychology, University of Nigeria, Nsukka, Nigeria; 2 Department of Health and Physical Education, University of Nigeria, Nsukka, Nigeria

**Keywords:** Gambling passion, Harmonious gambling passion, Obsessive gambling passion, Smartphone addiction, Schoolwork engagement

## Abstract

There are growing concerns that seem to suggest that students no longer engage in school-related activities as they ought to. Recent observation has revealed that students now spend excessive time participating in Internet gambling with their smartphone during school period. This trend could have far-reaching consequences on their schoolwork engagement and by extension, academic performance. Drawing on the Dualistic Model of Passion, this study therefore, examined the mediatory role of smartphone addiction in the gambling passion—schoolwork engagement relation. A cross-sectional design was adopted. Male undergraduates (*N* = 278) of a large public university in Nigeria who engage in Internet gambling participated in the study. They completed self-report measures of gambling passion, smartphone addiction, and schoolwork engagement. Results showed that harmonious gambling passion was not related to smartphone addiction whereas it was positively related to schoolwork engagement. Obsessive gambling passion had positive and negative relations with smartphone addiction and schoolwork engagement, respectively. Smartphone addiction was negatively related to schoolwork engagement and mediated only the obsessive gambling passion—schoolwork engagement relation but not that between harmonious gambling passion and schoolwork engagement. The theoretical and practical implications of the findings are discussed.

## Background

Schools represent contexts where students attend classes, work on projects and assignments, and study (Salanova et al. 2010; Salmela-Aro and Upadaya 2012). They are also places where students strive toward accomplishing specific goals such as completion of course work, academic performance, acquiring a degree and the like (Siu et al. 2014). However, there are indications that students’ interest in their schoolwork appears to be waning (Appleton et al. 2008). Insinuations suggest that the chances of students disengaging from their schoolwork increases as they progress from lower to higher levels of their education (Marion et al. 2014; Siu et al. 2014). If students are however fully engaged in their schoolwork, it could portend positive outcomes for them. Conversely, if they are not, their academic achievement could be undermined. For instance, several studies have linked increased levels of schoolwork engagement to positive outcomes such as increased performance (e.g., Bakker et al. 2015; Salanova et al. 2010; Schaufeli et al. 2002) whereas other studies linked lower levels of schoolwork engagement to a range of negative outcomes including increased school burnout and depressive symptoms (e.g., Marion et al. 2014; Salmela-Aro et al. 2009; Salmela-Aro and Upadaya 2012). Taken together, these studies are indicative of the importance of engaging in schoolwork in the lives of students. In essence, schoolwork engagement refers to energy, dedication toward, and absorption in schoolwork (Salmela-Aro and Upadaya 2012). Energy herein is characterized by positive approach to schoolwork. Dedication refers to a strong positive cognitive attitude and perceiving schoolwork as worthwhile. Absorption is characterized by being fully concentrated on studying, whereby time seems to pass quickly.

In the light of the apparent importance of schoolwork engagement, it would seem important to consider the factors that could impact on schoolwork engagement as well as the psychological processes that may underpin such impact. In that sense, one possible antecedent that is purported, in the current study, to have an impact on undergraduates’ schoolwork engagement is gambling passion. This follows our recent observation that during class and off class periods, male undergraduates in particular, are seen engaging in Internet football betting/gambling. A similar observation was made by Lo et al. (2005) who decried that students now spend substantial amount of time on regular basis playing video games in cybercafés rather than engaging in school-related activities.

Consistent with our observation, empirical evidence reveals that this trend is particularly common among males than females. For instance, Husky et al. (2015) recently found that women were less likely to engage in multiple gambling activities in comparison to men, while men are three times more likely to experience problems with gambling as compared to women. Tsitsika et al. (2011), reported that more males engage in gambling than females. For instance, a large cross-cultural study conducted by Peltzer and Pengpid (2014) involving 17,789 university students from 23 countries across Africa, Asia and Americas, showed that in Nigeria (*n* = 424), 13.8 % of the students reported gambling less than once a week whereas 6.7 % reported gambling once a week or more. In terms of gender, 10.1 % of men reported gambling once a week or more as compared to only 2.7 % of women who reported doing so. Across the countries, 4.6 % of men reported betting on sports once a week or more as compared to only 0.9 % of women who reported doing so. Furthermore, studies have also linked gambling and gambling passion to detrimental health consequences among undergraduates (e.g., Skitch and Hodgins 2005; Stuhldreher et al. 2007). Moreover, a 75 % rate of gambling participation has been reported among university students (e.g., Barnes et al. 2010). These trends appear to highlight the need to study gambling passion among male university students.

Indeed, as Vallerand and colleagues posited (Vallerand et al. 2007), “… being passionate for an activity leads individuals to dedicate themselves fully to their activity, thereby allowing them to persist, even in the face of obstacles, and to eventually reach excellence” (p. 506). Even though passion may engender dedication toward the activity in question and, in the long run, performance (Vallerand et al. 2007), it is possible that it may also enhance or undermine schoolwork engagement depending on the particular type of gambling passion (i.e., harmonious or obsessive gambling passion) that is adopted. Thus, given the preceding evidence that seem to suggest that gambling passion could have far-reaching consequences on undergraduates’ schoolwork engagement, it is however surprising to note that we have not come across any published studies that have examined the relationship between gambling passion and schoolwork engagement.

Moreover, our recent observation revealed that undergraduates who gamble on the Internet sometimes do so using their smartphones. Similarly, previous research has also indicated that problem gamblers are more likely to use a cell phone to gamble on the Internet than social gamblers (e.g., McBride and Derevensky 2009). In that vein, it is possible for smartphone addiction to mediate the influence of gambling passion on schoolwork engagement. In other words, having passion for Internet football betting/gambling may lead to smartphone addiction due to attempt at keeping track of ongoing/live football gambling on the Internet, which in turn may affect the schoolwork engagement of undergraduates. Again, heretofore, no published studies to our knowledge have examined the link between smartphone addiction and schoolwork engagement.

Several disparate studies have been conducted on schoolwork engagement under different but related appellations including but not limited to study engagement, student engagement and the like (e.g., Bakker et al. 2015; Marion et al. 2014; Salmela-Aro et al. 2009; Salmela-Aro and Upadaya 2012; Schaufeli et al. 2002). The literature is also replete with studies on Internet gambling, problem gambling, pathological gambling, and gambling passion (e.g., Barrault and Varescon 2013; Li et al. 2012; Ratelle et al. 2004; Tsitsika et al. 2011), as well as smartphone addiction (e.g., Al-Barashdi et al. 2015; Kwon et al. 2013a, b; Mok et al. 2014). However, to our knowledge, no published research has examined gambling passion as an antecedent of schoolwork engagement and the potential mediatory role of smartphone addiction in this relation. That is, there has not been any synthesis of these constructs within the same study. This lack of research synthesis impedes our understanding of how gambling passion and smartphone addiction can affect schoolwork engagement among undergraduates. Nonetheless, it seems probable that undergraduates who are passionate about football gambling may become addicted to their smartphone in order to sustain their passion for the gambling online, which in turn, may lead to lower levels of engagement in their schoolwork as well.

### Gambling and smartphone addiction: the Nigerian scenario

In Nigeria, the National Lottery Regulatory Commission (NLRC) is the body saddled with the responsibility of regulating lottery/gambling operations in order to promote transparency and accountability as well as protecting the interests of players, stakeholders and the public at large. It was established by the National Lottery Act (2005). Legally permitted lottery/gambling operations by the commission are: charitable lottery, lottery concierge services, online lottery, promotional lottery, short-message service (SMS) lotteries, sports lottery, USSD lotteries, and others. Thus, Internet football betting/gambling, which is the focus of the present study, by default falls under sports lottery (betting). The NLRC referred to sports betting as any activity involving prediction of sporting results and placing a bet on its outcome in anticipation of winning a set prize. All organizations currently operating the business of sports lottery (betting) and intending ones in Nigeria are required to obtain a Sports Lottery Permit (SLP) from the commission. The Sport Lottery Operator (SLO) must ensure that only those who are 18 years old and above are allowed to wager (for details, see the National Lottery Act 2005). Sports betting centres are located across the landscape of both remote and major cities in Nigeria. In most instances, such centres also serve as match viewing centres, where football fans come to watch their favourite football teams play. Bettors can bet with as low as even ₦100 in shops, over the phone and even online. All these indicate that Internet football betting/gambling is thriving in Nigeria.

In addition, Nigeria is part of the global users of smartphone. Globally, it has been reported that Nigeria is ranked 17th among countries who like using smartphones (Ekpeke 2015). In a recent submission, eMarketer (2015), a digital market analytical platform, estimated that the number of smartphone users in Nigeria will increase from 18.7 % in 2015 to 27.6 % by 2019. Furthermore, a recent study involving science students from privately owned universities in Nigeria revealed that majority of the students (83.7 %) enjoyed using their smartphones as compared to other mobile devices (Fasae and Adegbilero-Iwari 2015). In another Nigerian study, 97 % of undergraduates reported that they own a mobile phone (Okafor and Malizu 2014). Our observation also reveals that most students are seen on campus with their smartphones clutched at their hands. Taken together, these indicate that undergraduates in Nigeria are active users of smartphone.

## The present study

**Fig. 1:**
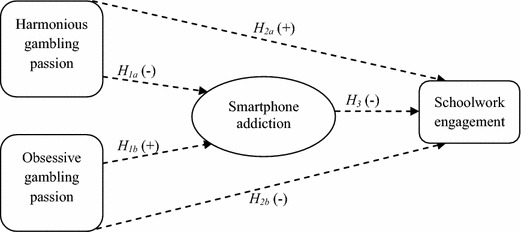
Proposed theoretical model of the relationship between gambling passion, smartphone addiction and schoolwork engagement

We aim to bridge the gaps in the literature by investigating smartphone addiction as the psychological process that could help explain the probable differential impact that the two types of gambling passion (i.e., harmonious and obsessive gambling passion) could have on schoolwork engagement. A theoretical model is proposed (see Fig. 1) based on the Dualistic Model of Passion (Vallerand et al. 2003) such that harmonious gambling passion is expected to be negatively related to smartphone addiction, whereas obsessive gambling passion is expected to be positively related to it. Smartphone addiction should in turn be associated with poorer schoolwork engagement. Thus, this study has the potential to contribute to the emerging schoolwork engagement literature by investigating gambling passion as a previously unexplored antecedent of schoolwork engagement as well as smartphone addiction as a mediating mechanism that could help account for this relationship. At the same time, it could aid in designing assessment and intervention strategies that will be useful in uncovering factors that could affect students’ schoolwork engagement as well as ways of improving such engagement.

## Theoretical background and hypotheses development

Vallerand et al. (2003) defined passion as “a strong inclination toward an activity that people like, that they find important, and in which they invest time and energy” (p. 757). In their Dualistic Model of Passion, Vallerand et al. (2003) posit that passion represents a major motivational force behind participation in enjoyable activities by providing people with the required energy to engage in pleasurable activity. It is this pleasurable activity that is expected to lead to different outcomes. The Dualistic Model of Passion can aid in the understanding of gamblers’ motivation in participating in gambling activities. According to this model, being passionate about an activity can be so self-defining such that it embodies key characteristics on the person’s identity and thus used as criteria for defining the person. For instance, individuals who are passionate about gambling do not merely gamble; they are ‘gamblers’. In other words, passionate gambling has become internalized into their identity.

The Dualistic Model of Passion (Vallerand et al. 2003) distinguishes between two types of passion that could develop based on the type of internalization process that takes place (Deci and Ryan 2000; Ryan and Deci 2000). They are: harmonious and obsessive passion. Harmonious passion stems from autonomous internalization of an activity into a person’s identity which occurs when a person has willingly accepted the activity as important and without any external rewards attached to it (Vallerand et al. 2003). There is no conflict between the enjoyable activity and other domains of the person’s life. Although the passionate activity occupies a considerable, but not overriding space in the person’s life, such that the person still remains in control while engaging in the enjoyable activity and is in harmony with other areas of the person’s life (Vallerand et al. 2003). In this respect, the Dualistic Model of Passion would suggest that undergraduates with harmonious passion for gambling, even though they are passionate about the gambling, are still in control of the gambling and thus, will not allow the gambling to override other domains of their lives such as schoolwork engagement.

While the football match is going on, gamblers are usually presented with different options or odds of winning on the Internet. Thus, given our recent observation and empirical evidence (e.g., Fasae and Adegbilero-Iwari 2015) which indicate that most undergraduates now have smartphones in Nigeria, one way that those who engage in Internet football gambling among them now choose to follow-up this gambling is to use their smartphones. This affords them the opportunity to keep abreast with the best possible options that could enhance their chances of winning. Consistent with the Dualistic Model of Passion (Vallerand et al. 2003), even though undergraduates with harmonious passion toward gambling may choose to use their smartphones for Internet gambling purposes, they are unlikely to become addicted to it at the detriment of engaging in their schoolwork. This is because they are in control of the gambling situation and gamble primarily for autonomous reasons (e.g., fun) and not necessarily because of external benefits (e.g., monetary reward). Hence, the Dualistic Model of Passion would suggest that undergraduates with harmonious passion for Internet football gambling are less likely to become addicted to their smartphone and in turn should engage more in their schoolwork.

In contrast to harmonious passion, obsessive passion originates from a controlled internalization of an enjoyable activity into the person’s identity. Intra- and/or interpersonal pressure is said to underlie such controlled internalization because there are certain benefits derived from the passionate activity, such as feeling socially accepted or worthy, or because the pleasure derived from the activity can no longer be controlled by the individual (Vallerand et al. 2007). Consequently, the passionate activity may become too valued, to be giving preference over all other activities in the person’s life, and to occupy so much space in the person’s identity such that it interferes with other domains in the person’s life (Vallerand et al. 2003, 2007). Such compelled engagement should prevent an undergraduate with an obsessive passion toward gambling from completely focusing on the task at hand, and may interfere with other domains of their life such as schoolwork engagement.

Moreover, because of the nature of the gambling that is being done on the Internet, it is expected that undergraduates who are obsessed about Internet gambling will use their smartphones to access the gambling in order to stay abreast of the gambling. Eventually, it may get to a point when they become addicted to their smartphones given that it was internalized into their identity based on controlled or external contingencies (e.g., monetary gain). In turn, they may experience psychological dependence or addiction to their smartphones which is likely to impinge on their schoolwork engagement.

Figure 1 depicts our proposed theoretical model. The dashed lines and the signs are used to represent hypothesized relations such that harmonious and obsessive gambling passions are expected to have respective negative and positive relations with smartphone addiction. Smartphone addiction in turn is expected to have a negative relation with schoolwork engagement. Harmonious and obsessive gambling passions are also expected to have respective positive and negative relations with schoolwork engagement.

### Gambling passion and smartphone addiction

Recent advances in Information and Communication Technology (ICT) have come to bear on the way modern smartphones are designed to function. Smartphones are designed nowadays to suit our personal lifestyles and has revolutionized the way we communicate, do business, have fun, conduct research, and even work. Given the convenience, attractive features, and broad range of functions that can be performed with smartphones, many people are keen on acquiring them (Kwon et al. 2013a, b). For example, as at 2010, the number of smartphones purchased across the globe rose to 130 million and it was further projected that mobile devices will be the basic gadget for connecting to the Internet before 2020 in place of other traditional means of doing so (MacCormick et al. 2012). Hence, because smartphones are Internet-enabled, it is possible for its users to become addicted to them which may trigger physical and social problems just like those associated with Internet addiction (Shaw and Black 2008).

Drawing from the definition of Internet addiction offered by Weinstein and Lejoyeux (2010), we define smartphone addiction as the excessive or irresistible compulsion regarding smartphone use that leads to impairment in psychological and social functioning. The smartphone “addicts may use … [their smartphones] … for extended periods, isolating themselves from other forms of social contact, and focus almost entirely on the[ir] … [smartphones] rather than broader life events” (Weinstein and Lejoyeux 2010, p. 277). Smartphone addiction is a form of technological addiction (Lee et al. 2014) and as such is regarded as a non-chemical behavioural addiction involving human–machine interaction (Griffiths 1996, 2000). It is characterised by increasing usage tolerances, withdrawal symptoms, salience, mood changes, conflict and relapse (Griffiths 1996, 2000). In other words, smartphone addicts can be said to be unable to do without their smartphone and may find it extremely difficult to function without it. As such, it is possible that they may become depressed and isolated when they abstain from using their smartphone. They may also be mostly preoccupied with smartphone use by devoting so much time than is required to its use rather than other life activities.

What’s more, given the constant evolving of the gambling industry in terms of accessibility, affordability, availability, anonymity, convenience, endorsement, glamorization, and promotion through the Internet, many youths have taken to Internet gambling (Griffiths 2003; Messerlian et al. 2004; Monaghan et al. 2008). Such Internet gambling practices have been linked to a range of adverse health consequences for individuals who indulge in them (Griffiths et al. 2009; Jacobs 2004; Lloyd et al. 2010). This is because they are at risk of becoming addicted (Pallanti et al. 2006) even though they often see themselves as invulnerable and deny the probable negative outcomes of gambling (Derevensky et al. 2003). Hence, undergraduates constitute a potential group that could develop addictive behavioural patterns, especially smartphone addiction because they make the most use of smartphone services (Head and Ziolkowski 2012) especially in respect to surfing the Net and exploring applications which provide thrilling functions (Al-Barashdi et al. 2015). Besides, recent research (e.g., Mok et al. 2014) has reported smartphone addiction among university students which appear to corroborate our recent observation that revealed that university students use smartphone to engage in Internet football gambling.

If that be the case, are all undergraduates who are passionate about Internet football gambling and use their smartphone to take part in it likely to become addicted to their smartphones? Prior studies (e.g., Mageau et al. 2005; Rousseau et al. 2002; Vallerand et al. 2003, 2007) suggest that it should be so only for those who are obsessively passionate about gambling. For instance, in two studies, Vallerand et al. (2007, Study 1 and 2) demonstrated that harmonious passion was positively related to subjective well-being (SWB) whereas obsessive passion was either not related or negatively related to SWB among dramatic arts students from different theatre schools and colleges across the Province of Quebec. In another study on background and consequences of different styles of engagement in video game play, Przybylski et al. (2009) found that low levels of basic need satisfaction were related to higher obsessive passion, higher amounts of play, more tension after play, and diminished game enjoyment, whereas higher levels of need satisfaction were not related to hours of play but were related to increased harmonious passion, game enjoyment, and energy after play. Further, Ratelle et al. (2004) found that being obsessively passionate about gambling was associated with a range of negative outcomes (e.g., poorer vitality and concentration in daily tasks, as well as increased rumination, anxiety, negative mood, guilt, and problem gambling) among participants recruited at the Montréal Casino, whereas such was not so for harmonious passion. Similar pattern of results were also obtained by Mageau et al. (2005) among those also involved in casino activities. In their investigation of the social-motivational antecedents of passion, Mageau et al. (2009) found that children and teenagers that were in autonomy-supportive environment were more likely to develop harmonious passion than obsessive passion. Meanwhile children and teenagers who attached high value to activity specialization, their activity are self-defined, and their parents exceedingly value the activity are more likely to develop obsessive than harmonious passion. Most revealing is a recent meta-analysis by Curran et al. (2015) involving 94 independent studies on the intrapersonal correlates of harmonious and obsessive passion. The results showed that harmonious passion was positively associated with positive intrapersonal outcomes (e.g., positive affect, satisfaction, flow, performance) and also had either non-significant or negative relationships with maladaptive intrapersonal outcomes (e.g., negative affect, performance avoidance goals and activity/life conflict). On the contrary, obsessive passion had a less desired and sometimes maladaptive pattern of relationships with both positive and negative intrapersonal criterion variables (e.g., negative affect, rumination, vitality). Remarkably, the effect sizes for the positive relationships between obsessive passion and adaptive intrapersonal outcomes (e.g., well-being and integrated motivation regulation) were significantly smaller in magnitude (small-to-moderate) in comparison to harmonious passion (moderate-to-large). Moreover, the previously significant positive associations between obsessive passion and these adaptive outcomes were reduced to non-significance when harmonious passion was controlled for. However, all positive relationships between obsessive passion and maladaptive outcomes remained significant while controlling for harmonious passion.

Based on evidence from previous studies on passion, we contend that partaking in gambling may not inevitably lead to negative outcomes. Rather, negative consequences following the gambling involvement are likely to ensue only if obsessive passion is involved. However, gambling that stems from harmonious passion may even lead to some positive consequences. Therefore, we propose that harmonious passion toward gambling should be negatively related to smartphone addiction whereas obsessive passion toward gambling should be positively related to smartphone addiction.

#### **Hypothesis 1a**

Harmonious passion toward gambling will be negatively related to smartphone addiction.

#### **Hypothesis 1b**

Obsessive passion toward gambling will be positively related to smartphone addiction.

### Gambling passion and schoolwork engagement

Success in school is generally desired by most individuals. Yet for students to be successful in their academic work, they need to be engaged in their schoolwork. Previous studies already indicated that factors such as maternal affection, friends (Marion et al. 2014), and achievement-related personal goal (Vasalampi et al. 2009) may influence students’ schoolwork engagement. However, we add to that list by submitting that schoolwork engagement can be influenced by gambling passion for several reasons. First, the entry into university signify a critical turning point for undergraduates because of the opportunity it affords them to leave home and socialize with their peers away from the prying eyes of their parents. This then appears to give them the leeway to get involved in not just positive behaviours but also negative ones. Supporting this stance, research has shown that university period is usually a time when students indulge in high-risk behaviours (Stuhldreher et al. 2007). Second, there are strong indications that students expend more time playing video games in cybercafés than in school-related activities (Lo et al. 2005). Third, we have observed that undergraduates constitute considerable number of fans of various football clubs across the world to the extent that they take part in Internet football wagering. Lastly, there are also strong evidences linking gambling and gambling passion to adverse health consequences among undergraduates (e.g., Skitch and Hodgins 2005; Stuhldreher et al. 2007). Collectively, these evidences suggest that gambling passion should influence schoolwork engagement. However, gambling passion may have different impacts on criterion variables depending on the particular type of passion that is at play (Curran et al. 2015; Vallerand et al. 2003). For example, undergraduates with harmonious passion toward gambling, even though they indulge in gambling, seem to understand the importance of engaging in their schoolwork because they are in control of the gambling. Hence they would not want their passion toward gambling to interfere with their schoolwork engagement. In contrast to harmonious passion, those with obsessive passion toward gambling have uncontrollable urge to indulge in the gambling. That is, gambling takes precedence over other activities in their lives and such individuals may experience negative consequences due to the gambling. For such undergraduates, the negative consequences may include poor academic performance; loss of money that was meant for school fees, feeding and other sundry expenses; insufficient sleep due to having to stay awake at night in order to follow-up the gambling (Stinchfield et al. 2006) especially if the need arises; anxiety over the amount of money invested in the gambling which could later turn into depression if huge sums of money are lost, etc. In line with Vallerand et al.’s (2003) proposition, such individuals with obsessive passion toward gambling could end up feeling guilty for expending too much time on gambling in place of engaging in their schoolwork.

Indeed, previous studies seem to provide support for the notion that harmonious passion should increase schoolwork engagement whereas obsessive passion should decrease it. More importantly, studies on passion have uncovered the effects of having either a harmonious or obsessive passion toward an activity that individuals are passionate about. For instance, research (e.g., Carpentier et al. 2012) with college students between the ages of 17 and 32 years shows that harmonious passion is positively associated with psychological well-being indicators such as life satisfaction and flow in one’s studies, while being unrelated to ruminations. In turn, flow in the favourite activity was positively related to psychological well-being. On the contrary, obsessive passion was negatively related to psychological well-being but was unrelated to flow experiences during one’s favourite activity. Obsessive passion also predicted higher levels of ruminations which in turn, were negatively related to the experience of flow. Studies have also revealed that obsessive passion predicts pathological gambling whereas harmonious passion does not (Ratelle et al. 2004). Furthermore, Vallerand et al. (2003, Study 2) found that harmonious passion toward football was related to higher positive affect during the whole football season whereas engaging in the passionate activity out of obsessive passion was related to increase negative affect during the same period. These results have been replicated in ample number of studies including diary studies (Mageau and Vallerand 2007) and studies involving football fans (Verner-Filion et al. 2012). These findings suggest that harmonious passion involves positive feelings during activity engagement whereas obsessive passion entails negative feelings while participating in the same activity. Thus, gambling passion may affect undergraduates’ schoolwork engagement by engendering either positive or negative feelings depending on whether they engage in such activity out of harmonious or obsessive passion, respectively.

Generally, results from previous studies robustly indicated that harmonious passion leads to positive and desirable outcomes whereas obsessive passion leads to negative and less desirable outcomes. These findings make us to hypothesize that harmonious passion toward gambling should enhance schoolwork engagement whereas obsessive passion should reduce it.

#### **Hypothesis 2a**

Harmonious passion toward gambling will be positively related to schoolwork engagement.

#### **Hypothesis 2b**

Obsessive passion toward gambling will be negatively related to schoolwork engagement.

### Smartphone addiction as a mediator between gambling passion and schoolwork engagement

As we earlier noted, Internet gamblers sometimes use their smartphone to partake in gambling due to the nature of the gambling and the ease of access to the Internet which smartphone affords them. Thus being passionate about Internet football gambling could lead to smartphone addiction since smartphones are used for Internet gambling purposes. In turn, being addicted to smartphone could have far-reaching adverse consequences on schoolwork engagement especially for undergraduates because they constitute the highest number of individuals who consume smartphone services (Head and Ziolkowski 2012). In addition, because smartphones are Internet-enabled, there is the likelihood for its users to become addicted to them which may result to physical and social problems analogous to those associated with Internet addiction (Shaw and Black 2008).

Empirical evidence from earlier studies appears to support the idea that gambling passion should impact upon smartphone addiction. In turn, smartphone addiction should be related to less schoolwork engagement. In other words, it is possible for smartphone addiction to mediate the impacts of Internet football gambling passion on schoolwork engagement. For instance, in a study involving university students, Skitch and Hodgins (2005) found that problem gamblers reported higher levels of both obsessive and harmonious passion for gambling, and that obsessive passion toward gambling was associated with severity of problem gambling behaviour whereas harmonious passion was not. Studies have also linked obsessive passion to increased rumination whereas harmonious passion was not (e.g., Ratelle et al. 2004). This could also be taken to suggest that individuals who are obsessively passionate about Internet football gambling are likely to ruminate about their smartphone because it facilitates involvement in the gambling whereas those with harmonious passion for Internet football gambling should not. Other studies have also indicated that gambling generally is associated with undesirable outcomes. For example, Lloyd et al. (2010) provided evidence that revealed that Internet gamblers of different betting patterns (i.e., non-to-minimal gamblers, sports bettors, casino and sports gamblers, lottery players, and multi-activity gamblers) were linked to varied degrees of problem gambling, mood disorders, substance misuse, history of deliberate self harm, and mental disorder. Tsitsika et al. (2011) found that Internet gambling was associated with problematic Internet use among adolescents. In a study involving online gamblers, McBride and Derevensky (2009) found that problem gamblers are more likely than social gamblers to spend more time gambling with a cell phone. All in all, given the evidence reported in previous studies, one would also expect that Internet football gambling passion will impact on smartphone addiction because most smartphones function basically through Internet-based applications.

Without doubt, smartphones can be beneficial to us in many ways especially in areas of enhanced communication and interpersonal interaction. However, there are ongoing concerns on its addictive potential and the concomitant negative consequences. In this regard, Levine et al. (2007), for example, cautioned against the use of such phones basically for leisure rather than academic purposes because it can serve as a distraction to learning in an academic environment. Moreover, recent evidence suggests that excessive smartphone use can be problematic (e.g., Mok et al. 2014). Studies in academic setting have also established an association between cell phone use and academic performance. For example, a study by Lepp et al. (2014) showed that increased cell phone use/texting was associated with decreased GPA and increased anxiety in a large; Midwestern US public university. More recent studies have also provided evidence on the negative consequences of cell phone use among students. For example, in a sample of undergraduates from a large public university in the US, Lepp et al. (2015) showed that after controlling for sex, cigarette smoking, class standing, self-efficacy for self-regulated learning, self-efficacy for academic achievement, and actual high school GPA, cell phone use negatively predicted GPA.

So far, since past research suggest a possible link between Internet gambling and smartphone addiction (e.g., McBride and Derevensky 2009) and smartphone use/addiction has been repeatedly related to poor academic outcomes among university students (e.g., Lepp et al. 2014, 2015), we therefore would expect that smartphone addiction may undermine schoolwork engagement and also serve as an intermediary between gambling passion and schoolwork engagement among undergraduates.

#### **Hypothesis 3**

Smartphone addiction will be negatively related to schoolwork engagement.

#### **Hypothesis 4a**

The relationship between harmonious gambling passion and schoolwork engagement will be mediated by smartphone addiction.

#### **Hypothesis 4b**

The relationship between obsessive gambling passion and schoolwork engagement will be mediated by smartphone addiction.

## Methods

### Participants

A total of 278 male students of a large federal university in Nigeria who used smartphones to gamble participated in this study. Our choice of only male students follows our observation that only male students engage in Internet football betting/gambling in the context where the study was conducted. Besides, similar studies have equally revealed that more males participate in Internet gambling than females (e.g., Tsitsika et al. 2011) and as such, recent studies (e.g., Wang et al. 2011) have begun to focus exclusively on male participants. Participants were between the ages of 16 and 34 years, with a mean age of 22.39 years (*SD* = 3.30). All participants had engaged in Internet football betting/gambling at least once over the past month preceding data collection and own a smartphone.

### Procedure

Individuals who participate in Internet gambling were recruited as participants from three Internet football betting centres located close to a large federal university in Nigeria who came to wager on football. In total, we approached 328 males between May and August, 2015 and asked whether they are students of the federal university. The 319 individuals who identified themselves as students of the university were then asked if they own a smartphone and use it for gambling purposes. A total of 310 students used smartphones for gambling purposes. These students (310) were then asked to participate in a study aimed at gaining a better understanding of their gambling, smartphone, and school-related activities. Of the 310 students, 290 consented to participate and were thus handed a questionnaire that included the Gambling Passion Scale (GPS; Rousseau et al. 2002), Smartphone Addiction Scale-Short Version (SAS-SV; Kwon et al. 2013a), and Schoolwork Engagement Inventory (EDA; Salmela-Aro and Upadaya 2012) as well as demographic items on gender and age. All the two hundred and ninety (290) copies of the questionnaire distributed were completed and collected on the spot. However, only properly completed questionnaires (i.e., 278) were subjected to statistical analysis. The remaining 12 were discarded due to improper completion. All in all, the response rate was 95. 86 %.

### Measures

Participants completed self-report questionnaire assessing gambling passion, smartphone addiction, schoolwork engagement, and demographic information (gender and age). All response formats were based on Likert scales except demographic items.

*Schoolwork engagement* was assessed with the Salmela-Aro and Upadaya’s (2012) Schoolwork Engagement Inventory (EDA), which comprises nine items that measure energy (e.g., “I feel strong and vigorous when I am studying”), dedication (e.g., “My schoolwork inspires me”), and absorption (e.g., “I feel happy when I am working intensively at school”) in relation to schoolwork. Respondents rated the degree to which each item applied to them based on estimation from the previous month, using a 0 (*never*) to 6 (*daily*) response scale. Items were averaged to form an overall scale score. Higher scores indicate greater engagement with schoolwork. The inventory has satisfactory level of Cronbach’s alpha (α) coefficient of .77 in the present study.

*Gambling passion* was measured with the Rousseau et al.’s (2002) Gambling Passion Scale (GPS). Respondents were asked to think about their favourite Internet football betting/gambling games and then complete the items on the GPS with respect to this activity. The GPS consists of 10 items, 5 for each subscale: obsessive gambling passion (e.g., “I have almost an obsessive feeling for this gambling game”) and harmonious gambling passion (e.g., “This gambling game reflects the qualities I like about myself”). The responses are rated on a 7-point scale ranging from 1 (*not agree at all*) to 7 (*very strongly agree*). Possible score on the GPS ranged from 5 to 35 for each of the subscales. Each respondent’s scores were averaged to form a subscale score. Higher scores represent higher obsessive or harmonious passion toward gambling. Estimated Cronbach’s α coefficient of both obsessive and harmonious gambling passion subscales respectively, are .84 and .85 in the present study.

*Smartphone addiction* was measured with the Smartphone Addiction Scale-Short Version (SAS-SV) developed by Kwon et al. (2013a). The SAS-SV consists of ten items. Participants’ responses to each item were scored using a 6-point scale ranging from 1 (*strongly disagree*) to 6 (*strongly agree*). Sample items include: “Having my smartphone in my mind even when I am not using it”, and “The people around me tell me that I use my smartphone too much.” Possible score on the scale ranged from 10 to 60. Scores on each item were averaged to form an overall score for the SAS-SV. Higher scores represent greater addiction to smartphone. The SAS-SV is internally consistent with Cronbach’s α coefficient of .76 obtained in the present sample.

## Ethical considerations

We tried to address some ethical issues during the study period. For all prospective respondents, we explained the nature and purpose of the study orally to them. We also clearly stated, both orally and through a letter that accompanied the questionnaire, that participation was voluntary, anonymous, and confidential. Finally, we informed them that they were free to withdraw at any time in the study without any penalty.

### Strategy of data analysis

We conducted some preliminary analyses on the means, standard deviations, reliabilities, and correlations among all key variables using the Statistical Package for the Social Sciences (SPSS) version 21.0. The internal consistency reliability was estimated based on Cronbach’s alpha. Correlations are presented here to test whether the independent variables were too related (i.e., an indication of the risk of multicollinearity) that may warrant combining the scores of the variables to form a composite score. To test our main effects hypotheses, we conducted two hierarchical multiple regression with smartphone addiction and schoolwork engagement as outcome variables, respectively. To test our mediation effects hypotheses, we conducted bootstrapping. Because previous research has demonstrated that age can influence schoolwork and study engagement (e.g., Bakker et al. 2015; Marion et al. 2014), mobile phone use (e.g., Walsh et al. 2011), and gambling severity (e.g., McBride and Derevensky 2009), we controlled for this variable in the analyses. Further, due to conceptual similarities between harmonious and obsessive gambling passion, we controlled for each of them to ensure that our results were not spurious and misleading as well as to be able to detect the unique influence that each type of gambling passion exerts on smartphone addiction and schoolwork engagement. Similar approach was also adopted in a recent study (e.g., Curran et al. 2015).

## Results

### Preliminary analysis

Means, standard deviations, reliabilities (Cronbach’s alpha), and correlations matrix for all measured variables are presented in Table 1. Inspection of the Cronbach’s alpha coefficients showed satisfactory levels of internal consistency reliabilities of the scales that exceeded the cutoff rules-of-the thumb of .70 commonly recommended for research purpose (Kaplan and Saccuzzo 2013). Besides, the correlations among the study variables, ranging from −.06 to .27, indicated no threat of multicollinearity.

**Table 1:** Means, standard deviations, alpha coefficients, and correlations among the study variables

Variable	*M*	*SD*	1	2	3	4	5
1. Age	22.39	3.30	–				
2. Harmonious passion	28.14	2.87	−.23***	(.84)			
3. Obsessive passion	8.27	2.55	−.10	.11	(.84)		
4. Smartphone addiction	18.55	4.02	−.13*	.06	.27***	(.76)	
5. Schoolwork engagement	46.09	3.30	−.06	.18**	−.11	−.19***	(.77)

Cronbach’s alpha coefficients for the scales are on the diagonal of the matrix in parentheses

*N* = 278, * *p* < .05 (two-tailed); ** *p* < .01 (two-tailed); *** *p* < .001 (two-tailed)

The results in Table 1 indicated that age was significantly and negatively correlated with smartphone addiction (*r* = −.13, *p* = .033) but not significantly correlated with schoolwork engagement (*r* = −.06, *n.s.*). Harmonious passion was not significantly correlated with smartphone addiction (*r* = .06, *n.s.*) whereas it was significantly correlated with schoolwork engagement (*r* = .18, *p* = .003). Obsessive passion was significantly and positively correlated with smartphone addiction (*r* = .27, *p* < .001) but was not significantly correlated with schoolwork engagement (*r* = −.11, *n.s.*). Smartphone addiction was significantly and negatively correlated with schoolwork engagement (*r* = −.19, *p* = .001).

### Hypotheses testing

**Fig. 2:**
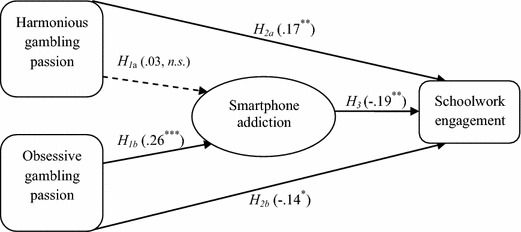
Results of hierarchical multiple regression of predictors of schoolwork engagement. *Note* *p < .05; **p < .01; ***p < .001. The *numerical values* on the *arrows* pointing to smartphone addiction are beta weights taken from the first regression while those pointing to schoolwork engagement are taken from the second regression

The results of the hierarchical multiple regression and bias corrected (BC) bootstrapping appear in Fig. 2 and Table 2, respectively. Bootstrapping is a re-sampling method computed in order to derive more precise confidence intervals (CI) that has advantage over the causal steps method of Baron and Kenny and the Sobel test that is based on the product of coefficients and the assumption that the sample must be normally distributed before mediation can occur (Hayes 2009; Preacher and Hayes 2004). These assumptions are not necessary in bootstrapping in that it can well be used on small sample size. Thus, given the inherent limitations in the Baron and Kenny’s approach as well as the Sobel test, researchers have begun to discourage the use of such procedures in mediation analysis (Hayes 2009; Preacher and Hayes 2004). We used the PROCESS for SPSS macro (version 2.13) to produce the bootstrapped indirect effects. We drew new samples (with replacements) from our original sample 1000 times and calculated the indirect estimates of the model based on 95 % CI. Mediation is demonstrated if the entire CI lies above or below zero. That is, a predictor *X* has an indirect effect on *Y* through the mediating variable *M* (Hayes 2009; Hayes and Preacher 2010; Preacher and Hayes 2004).

**Table 2:** Mediating effect of smartphone addiction in the gambling passion—schoolwork engagement relation

Indirect effect pathways	Estimate	BC 95 % CI	
		Lower	Upper
HGP **→** SA **→** Schoolwork engagement	−.004	−.020	.010
OGP **→** SA **→** Schoolwork engagement	−.034*	−.073	−.010

BC bootstrapping results were based on 1000 bootstrapped samples

*BC* bias corrected, *CI* confidence interval, *HGP* harmonious gambling passion, *OGP* obsessive gambling passion, *SA* smartphone addiction

* *p* < .05

The results of the hierarchical multiple regression in Fig. 2 indicated that after controlling for the influence of age (β = −.13, *p* < .05), harmonious gambling passion was not significantly related to smartphone addiction (β = .03, *n.s.*) whereas obsessive gambling passion was significantly and positively related to smartphone addiction (β = .26, *p* < .001). Therefore *H*_*1a*_ was not confirmed whereas *H*_*1b*_ was confirmed. Both harmonious (β = .17, *p* = .005) and obsessive (β = −.14, *p* = .024) gambling passion were significantly related to schoolwork engagement in positive and negative directions, respectively. Furthermore, smartphone addiction had a significant negative relation with schoolwork engagement (β = −.19, *p* = .002). Thus, *H*_*2a*_*, H*_*2b*_, and *H*_*3*_ were confirmed.

We also tested if smartphone addiction mediated the relationship between the two components of gambling passion (i.e., harmonious and obsessive gambling passion) and schoolwork engagement. The results showed that the BC bootstrap CI for the indirect effect of harmonious gambling passion on schoolwork engagement via smartphone addiction was not statistically different from zero (*H*_*4a*_; bootstrap estimate = −.004, lower CI = −.020, upper CI = .010, *n.s.*) whereas that of obsessive gambling passion on schoolwork engagement via smartphone addiction was statistically different from zero (*H*_*4b*_; bootstrap estimate = −.034, lower CI = −.073, upper CI = −.010, *p* < .05). Thus the mediating role of smartphone addiction was confirmed for obsessive gambling passion but not for harmonious gambling passion. That is, obsessive gambling passion was related to decreased schoolwork engagement through smartphone addiction.

## Discussion

The current study focused on gaining insight into how passion toward gambling relates to schoolwork engagement as well as in uncovering the psychological processes that might be responsible for the hypothesized opposing consequences that the two types of gambling passion (i.e., harmonious and obsessive gambling passion) may have on schoolwork engagement. Our expectations were partially supported as results revealed that harmonious passion toward gambling was unrelated to smartphone addiction (i.e., *H*_*1a*_ was not confirmed) whereas obsessive passion toward gambling was related to increased levels of smartphone addiction (i.e., *H*_*1b*_ was confirmed). These results suggest that having harmonious passion toward gambling may not lead to smartphone addiction, whereas the more individuals are obsessed about gambling, the more they seem to be addicted to their smartphone. Our results appear to be in line with the Dualistic Model of Passion (Vallerand et al. 2003), considering the possibility that individuals driven by harmonious passion toward gambling are in control of their gambling situation and expend great autonomy in participating in the gambling. As such, even if they use their smartphone for Internet gambling purposes, they may not become addicted to it. However, for those who are obsessively passionate about gambling, the reverse seems to be the case given that they are being driven by irresistible urge to indulge in the gambling which incidentally, using their smartphone makes easier for them. These findings are consistent with past research that also revealed that harmonious passion is either unrelated or negatively related to negative outcomes whereas obsessive passion is linked to increased negative outcomes (e.g., Carpentier et al. 2012; Curran et al. 2015; Mageau et al. 2005; Ratelle et al. 2004; Skitch and Hodgins 2005). These would explain why harmonious gambling passion was unrelated to smartphone addiction and the positive relationship found between obsessive gambling passion and smartphone addiction in the present study.

As expected, the results showed that both harmonious (*H*_*2a*_) and obsessive (*H*_*2b*_) gambling passion were related to schoolwork engagement and in the hypothesized directions (i.e., positive and negative, respectively). These findings suggest that having a harmonious passion toward gambling may also increase schoolwork engagement among undergraduates, whereas participating in the gambling activity out of obsessive passion seems to undermine their schoolwork engagement. These findings are consistent with the Dualistic Model of Passion (Vallerand et al. 2003) which posits that for those driven by harmonious passion; the passionate activity does not conflict with other aspects of the person’s life since the person is able to juggle successively between the passionate activity and other life domains. However, for those who are obsessed about gambling, they seem to be so preoccupied with the gambling, thereby leading to less attention being paid to their schoolwork. Indeed, consistent with past researches that have shown that having a harmonious passion toward an activity is linked to increased desirable outcomes such as psychological well-being indicators, mastery goal pursuit, performance and the like, whereas obsessive passion toward an activity is unrelated or negatively related to such desirable outcomes (e.g., Carpentier et al. 2012; Vallerand et al. 2007, 2008, Study 1 and 2), the current study shows that having a harmonious passion toward gambling is linked to increased schoolwork engagement whereas obsessive passion toward gambling is linked to decreased schoolwork engagement.

The results also provide evidence for *H*_*3*_ which predicted a negative relationship between smartphone addiction and schoolwork engagement. This finding suggests that increased smartphone addiction appears to lead to reduced levels of schoolwork among undergraduates. This finding is not surprising given that recent research has found that excessive smartphone/cell phone use has been linked to negative psychological health outcomes (e.g., Mok et al. 2014), and poor academic performance, especially among university students (e.g., Lepp et al. 2014, 2015). What this means in essence is that using smartphone to the extent of becoming addicted to it may lead to poorer schoolwork engagement among undergraduates. This would explain the negative relationship found between smartphone addiction and schoolwork engagement.

We expected that smartphone addiction would mediate the relationship between the two types of gambling passion and schoolwork engagement. However, our data only provided evidence for the mediatory role of smartphone addiction in the obsessive gambling passion—schoolwork engagement relation (*H*_*4b*_) but not for harmonious gambling passion—schoolwork engagement relation (*H*_*4a*_). These results suggest that smartphone addiction is an important avenue that helps in conveying the influence of obsessive gambling passion on schoolwork engagement. If undergraduates take part in Internet gambling out of obsessive gambling passion, they seem to become addicted to their smartphone because it serves as a medium of partaking in the gambling with ease, which in turn decreases their schoolwork engagement. Our results are consistent with previous studies that showed that obsessive passion was positively related to maladaptive outcome such as ruminations, which in turn was associated with decreased experience of flow (e.g., Carpentier et al. 2012). However, a plausible explanation as to why smartphone addiction did not mediate the relationship between harmonious gambling passion and schoolwork engagement could be because gambling based on harmonious passion may not necessarily lead to maladaptive outcomes as revealed in the present as well as past studies (e.g., Carpentier et al. 2012; Curran et al. 2015; Ratelle et al. 2004). Thus, maladaptive outcome such as smartphone addiction in turn may be unable to transmit the influence of harmonious gambling passion on schoolwork engagement in the present study. Supporting this stance, our earlier results (*H*_*1a*_) showed that harmonious gambling passion was unrelated to smartphone addiction in the first instance, and as such smartphone addiction could not convey/mediate the impact of harmonious gambling passion on schoolwork engagement.

## Limitations, strengths, and future directions

Any study of this nature has inherent limitations and needed to be mentioned. Therefore the findings of the present study must be interpreted in the light of these limitations. First, the study adopted a cross-sectional design and is correlational in nature such that the relationship between Internet gambling passion and smartphone addiction were tested in only one direction. We cannot completely rule out the possibility of a reciprocal relationship between Internet gambling passion and smartphone addiction such that smartphone addiction provides easy access to Internet gambling or whether Internet gambling passion leads to smartphone addiction. An important step for future research is to utilize longitudinal designs to determine whether changes in Internet gambling passion and smartphone addiction can account for significant variance in schoolwork engagement over a period of time. However, the method adopted in the current study is consistent with current approach (e.g., Carpentier et al. 2012).

Second, this study collected data based on only self-report measures. This may raise the issue of common method variance. Future studies should endeavour to utilize data from other sources such as the students’ peers and lecturers. However, we tried to limit the problem of common method variance by utilizing self**-**report measures with different response formats, ensuring anonymity and confidentiality of responses, and encouraging respondents to be honest in their responses (Podsakoff et al. 2003).

This study therefore contributes to the literature by extending the influence of gambling passion on an emerging construct (i.e., schoolwork engagement). The study also adds to the area of positive psychology by providing further support to the idea that having passion for an activity can also lead to positive outcomes in school setting depending on the type of passion that is adopted. Moreover, to our knowledge, this is the first study that has attempted to synthesize the relationships between gambling passion, smartphone addiction, and schoolwork engagement in a sample of male undergraduates within the same study. This study is also informative given that the data were gathered at an actual Internet football betting centres while undergraduates’ bettors were involved in actual Internet wagering activities.

## Theoretical and practical implications of findings

The findings of our study have some theoretical and practical implications. Indeed, we found evidence that the two types of gambling passion (i.e., harmonious and obsessive gambling passion) relate in different ways to common criteria—smartphone addiction and schoolwork engagement. This provides further support for the Vallerand et al.’s (2003) Dualistic Model of Passion that contends that engaging in passionate activity may not always lead to detrimental consequences, and can even be beneficial depending on the type of passion that is at play. In essence, even in academic setting, empirical evidence seems to favour the theory. Thus, the theory can well provide explanations as to why we found that harmonious and obsessive gambling passion related in different ways to smartphone addiction and schoolwork engagement.

From a practical view point, gambling has traditionally been viewed from a negative lens. For instance, Stuhldreher et al. (2007) have already cautioned that “Gambling is an emerging high-risk behavior that has sounded the alarm bell on campuses nationwide” (p. 75). However, as our findings suggest, such negative connotation usually comes into play especially when obsessive gambling passion is involved. Thus, rather than condemning or promoting gambling in its entirety, vocational counsellors and academic advisers should inform their students that it is possible for them to be passionate about gambling, while at the same time being highly engaged in their schoolwork activities, to the degree to which their passion for gambling is in harmony with other life pursuits, especially those that have to do with schoolwork. In other words, they can be passionate gamblers and still be able to strike a balance between their gambling activities and schoolwork engagements. They should also sensitize them on the dangers of being obsessively passionate about gambling as this may adversely interfere with their schoolwork engagement and could also impact negatively on other important school-related outcomes. School management should also endeavour to conduct regular screening exercises for gambling among their students using indices such as the revised South Oaks Gambling Screen (SOGS; Lesieur and Blume 1993) in order to identify those who are already or likely to become pathological gamblers. After the screening, they can then commence treatment for those who require such. Moreover, gambling regulatory agencies in Nigeria such as the National Lottery Regulatory Commission (NLRC) should focus on promoting responsible gambling activities. They should emphasize the need for gambling advertisements to clearly point out that gamblers should engage in gambling activities primarily for the fun of it rather than as a means of enriching themselves. They should also encourage the inclusion of pop-up messages in adverts during gambling on the Internet to occasionally remind Internet gamblers of the potential detrimental consequences of excessive gambling activities. Following our observation, the NLRC should also ensure that operators of gambling centres strictly comply with the gambling age of 18 years and above by penalising defaulters.

We also discovered that smartphone addiction seems to serve as an impediment to undergraduates’ schoolwork engagement. Although we do not advocate for the ban of the use of smartphone on campus given that it could also facilitate learning if used for learning purposes, we however concur with Levine et al. (2007) by urging smartphone users not to use such phones exclusively for leisure purposes on campus because it can be distractive to learning. Thus, school management could prevent or minimize the negative impact of smartphone addiction on their students by taking proactive steps at preventing or minimizing the occurrence of smartphone addiction through sensitization programmes on the dangers of being addicted to their smartphone. In a similar vein, conducting regular screening exercises for smartphone addiction using indicators such as the Smartphone Addiction Scale-Short Version (SAS-SV; Kwon et al. 2013a) not just for newly admitted students, but also for those admitted earlier could be beneficial. Such screening exercises have the potential in diagnosing students who are likely to become addicted or those already addicted to their smartphone and thus preventive measures or prompt commencement of treatment as the case may be, can ensue. In addition, given the apparent similarities between smartphone addiction and Internet addiction, some of the interventions designed for Internet gambling addiction purposes such as those highlighted in Griffiths (2003) could also be effective in preventing and alleviating smartphone addiction among university students.

## Conclusion

This study aimed at studying how the two types of gambling passion— harmonious and obsessive gambling passion relate to schoolwork engagement and whether smartphone addiction mediates these relations among a sample of undergraduates in Nigeria. The study was guided by the Dualistic Model of Passion (Vallerand et al. 2003) which contends that passion can lead to different consequences depending on the specific type of passion that is at play. Indeed, the results support the central proposition of the Dualistic Model of Passion, and thus broaden our understanding of the two types of gambling passion and their relationship with schoolwork engagement for undergraduates involved with Internet football gambling. It also offers additional and interesting insight into the mediating role of smartphone addiction. Thus, besides being associated with poorer schoolwork engagement, we also found strong evidence for the mediating role of smartphone addiction in the relationship between obsessive passion toward gambling and schoolwork engagement. Nonetheless, the findings of the current study call for prospective research to investigate other potential intervening variables, including but not limited to educational level, academic self-efficacy, gamblers’ beliefs, gambling motives, and gambling expectancy, in the association between gambling passion and schoolwork engagement in a more representative sample of not just university students but also secondary school students.
